# Metal Sulfide Nanoparticles for Imaging and Phototherapeutic Applications

**DOI:** 10.3390/molecules28062553

**Published:** 2023-03-10

**Authors:** Aishwarya Shetty, Heinrich Lang, Sudeshna Chandra

**Affiliations:** 1 Journal of Visualized Experiments 625, Massachusetts Avenue, Cambridge, MA 02139, USA; 2 Chemnitz Research Group Organometallics, MAIN Research Center, Technische Universität, Rosenbergstr. 6, 09126 Chemnitz, Germany; 3 Institute of Analytical Chemistry, University of Regensburg, 93040 Regensburg, Germany

**Keywords:** metal sulfide nanoparticles, bioimaging, photothermal therapy, photodynamic therapy, immunotherapy

## Abstract

The intriguing properties of metal sulfide nanoparticles (=MxSy-NPs), particularly transition metal dichalcogenides, are discussed for their use in diverse biological applications. Herein, recent advances in MxSy-NPs-based imaging (MRI, CT, optical and photoacoustic) and phototherapy (photothermal and photodynamic) are presented. Also, recent made progress in the use of immuno-phototherapy combinatorial approaches in vitro and in vivo are reported. Furthermore, challenges in nanomaterials-based therapies and future research directions by applying MxSy-NPs in combinatorial therapies are envisaged.

## 1. Introduction

In recent years, applications of nanotechnology have expanded into different branches of the biomedical field [1,2,3]. Efforts are continually being made towards the development of unique nanoparticles (=NPs) which can overcome limitations of traditional therapeutics and, hence, are able to improve management of diseases [4]. Large surface area-to-volume ratios of NPs provide a platform for easy chemical functionalization for excellent interaction with biological systems. Among the broad range of NPs studied for biomedical applications, metal sulfide nanoparticles (=M_x_S_y_-NPs) have been the focus of several studies in recent years [5,6,7]. In addition to properties found at the nanoscale, M_x_S_y_-NPs also exhibit favorable properties such as light conversion, Fenton catalysis, immune activation and radiation enhancement [8,9]. The lower electronegativity of sulfur in comparison to oxygen makes M_x_S_y_-NPs naturally versatile in comparison to highly exploited metal oxide ones [10]. The versatility of M_x_S_y_-NPs becomes evident by the fact that they can be successfully used for various applications including different types of imaging and therapy, often alone or in combination with other materials to enhance their intended application [11]. In addition, M_x_S_y_-NPs possess the ability to impart multiple functionalities as “stand-alone” systems without addition of other materials. For example, transition metal dichalcogenide-based molybdenum disulfide (MoS_2_-) and tungsten disulfide (WS_2_-) NPs are increasingly found in theranostic and biosensing applications [12,13]. Tunable bandgap and strong spin-orbit coupling make MoS_2_-NPs particularly interesting for biomedical applications, whereas strong near-infrared (NIR) absorptions has led to the efficacious use of copper sulfide (CuS-) NPs as photothermal agents [14,15].

Hence, herein, various uses of M_x_S_y_-NPs towards the above-mentioned background will be discussed on selected examples.

## 2. Applications of Metal Sulfide Nanoparticles in Bioimaging

### 2.1. Magnetic Resonance Imaging

As a result of the use of non-ionizing radiation, high spatial resolution and non-invasive magnetic resonance imaging (=MRI) has become one of the most used imaging techniques in the medical field [16]. MRI makes use of pulsed magnetic waves to align protons present in water and images are produced by recording radio-waves released by these protons upon their relaxation to the ground state [17]. Contrast agents are applied to significantly improve resolution and work by reducing the longitudinal or transverse (i.e., T_1_ or T_2_) relaxation time of protons in water [18]. Studies on NPs for MR imaging have mostly focused on metal oxides such as superparamagnetic iron oxide NPs (SPIONs); however, in recent years, researchers have begun exploring M_x_S_y_-NPs as well [19,20]. Examples of such studies reporting the use of M_x_S_y_-NPs, wherein the MR contrast is brought about by the metal sulfide itself, are highlighted below.

**Figure 1:**
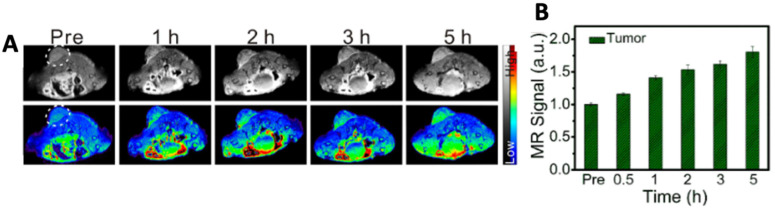
Representative images of (**A**) MR imaging and (**B**) its quantitative estimation. Reprinted with permission from Ref. [22]. Copyright © 2023, Elsevier.

Iron sulfide quantum dots (=FeS QDs) were synthesized via a biomimetic route using protein bovine serum albumin (=BSA) as a template. Nanoparticles based on FeS exhibit physicochemical properties similar to that of iron oxide nanoparticles as sulfur and oxygen are congeneric elements. However, iron sulfide (FeS, Fe_1−x_S, FeS_2_, Fe_3_S_4_) exist in more phases than iron oxide (Fe_3_O_4_, Fe_2_O_3_) showing more variability and also have a smaller band gap. The authors observed a strong NIR absorption which was exploited for photoacoustic imaging, whereas quantum confinement effects enabled fluorescence imaging. The longitudinal relaxation (=r_1_) value of FeS QDs (5.35 mM^−1^ s^−1^) was found to be higher than that of corresponding aggregates (0.2 mM^−1^ s^−1^), which is attributed to the template-assisted synthesis [21]. The resulting QDs thus showed good dispersion, higher longitudinal relaxivity, extended rotational correlation time and lower magnetization in comparison to the clinically used gadolinium-based MRI contrast agent Gd-DTPA (r_1_ = 3.1 mM^−1^ s^−1^). As observed in Figure 1, the authors tested the MR, PA and fluorescence imaging ability of FeS QDs in vivo in 4T1 tumor-bearing mice post-intravenous (i.v.) administration [22]. As can be seen in Figure 1A,B, MR contrasts at 5 h post-administration is 1.8-fold higher as compared to pre-administration.

A nanohybrid (=NH), based on the sulfides of bismuth and iron was prepared by Xiong et al. via biomineralization using BSA to yield Bi_2_S_3_/FeS_2_@BSA NHs [23]. BSA acted as a source of sulfur, as a template for the synthesis and as a reducing agent, whereas Fe and Bi provided the contrast for MR and computed tomography (=CT) imaging, respectively. The X-ray absorption coefficient of the NHs is 8.02 HU mM^−1^ which increased in proportion to increasing concentrations of Bi. A similar trend was observed for MRI contrast and r_2_, i.e., transverse relaxivity time was determined to 53.9 mM^−1^ s^−1^. In vivo, Bi_2_S_3_/FeS_2_@BSA NHs showed accumulation in the tumor with good CT and MR imaging contrast when injected intravenously in a 4T1 tumor-bearing mice [23]. Fu et al. exploited magnetocaloric and MR imaging properties of iron sulfide for imaging-guided thrombolysis in celiac vein thrombosis. The author’s synthesized hydrophilic polyvinyl pyrrolidone-capped Fe_3_S_4_-NPs with an r_2_ value of 53.1 mM^−1^ s^−1^ [24]. Through simultaneous exposure to an alternating magnetic field (=AMF) and an 808 nm laser, the NP dispersion attained a temperature higher than when exposed to AMF or laser alone. In vitro, the synergistic thermal conversion resulted in near disappearance of the thrombus, whereas individual stimulation resulted in partial dissolution. When tested in a C57 mice model of deep vein thrombosis, it resulted in the reduction of thrombus, which was visualized by MR imaging. Unpaired 3D electrons in cobalt (Co) were utilized by Lv and colleagues for T_2_-weighted MRI [25]. Therefore, the authors prepared hollow cobalt sulfide (Co_3_S_4_-) NPs which were coated with a shell of N-doped carbon and encapsulated the drug doxorubicin for therapeutic (chemotherapy, photothermal therapy and photodynamic therapy) and imaging (MRI and thermal imaging) applications [26]. The respective NPs showed a concentration-dependent increase in MR and thermal imaging contrast. In vivo, when tested in H22 tumor bearing mice, the nanoparticles showed a good contrast as compared to pre-treatment. Huang et al. synthesized Cu_2__−*x*_S@MnS core-shell NPs in which the Cu_2−x_S-NPs are surrounded by a manganese sulfide (MnS) shell [27]. NIR absorption by CuS enabled photothermal treatment, whereas the presence of MnS facilitated light-triggered photodynamic therapy (PDT) and MRI. The NPs showed high photothermal conversion efficiency (47.9%) and ability to generate reactive oxygen species (=ROS) in the presence of hydrogen peroxide. With respect to MRI, T_1_ contrast increased in proportion to the concentration of manganese and an r_1_ value of 1.243 mM^−1^ s^−1^ was reported. Similarly, Chen et al. reported on the assembly of CuS-MnS_2_ nanoflowers for MRI-guided photothermal-photodynamic therapy [28].

### 2.2. Computed Tomography

In CT imaging, differential tissue thicknesses and X-ray attenuations are exploited to generate three-dimensional and cross-sectional images [29]. High X-ray absorption as a consequence of high atomic numbers has resulted in the application of bismuth (Bi) and tungsten as CT contrast agents [30,31]. PEGylated-WS_2_-NPs, i.e., polyethylene glycol (PEG)-coated tungsten disulfide NPs for CT-guided photothermal therapy (PTT) were prepared by Wang and colleagues [32]. The CT-imaging ability of the NPs was tested in 4T1 tumor-bearing mice using phosphate-buffered saline (=PBS)-treated mice as a control group. In conclusion, good photothermal stability and an effective use as CT contrast agents were reported. Similarly, Wang et al. introduced manganese dioxide (MnO_2_-) coated mesoporous polydopamine nanosponges (=MPDA NSs) embedded with WS_2_ nanodots (=ND), i.e., MPDA-WS_2_@MnO_2_ for multimodal imaging guided thermo-radiotherapy of cancer [33]. WS_2_ NDs and MPDA NSs enabled radio-sensitization and PTT in addition to contrast for CT and multi-spectral optoacoustic tomography (=MSOT), respectively. The MnO_2_ component provided MRI contrast and tumor hypoxia modulating properties. In all three imaging modalities, the contrast provided by MPDA-WS_2_@MnO_2_-NPs increased linearly with increasing concentration of the NPs. The authors reported a CT value of 35.3 HU L g^−1^ and a transverse relaxation value of 6.696 mM^−1^ S^−1^ at pH 6.5. Post intratumoral (=i.t.) and intravenous (=i.v.) administrations. In vivo, an 8- and 2.5-fold increase in signal intensity was observed for CT and MSOT imaging, respectively. Similar results were also observed for MRI.

**Figure 2:**
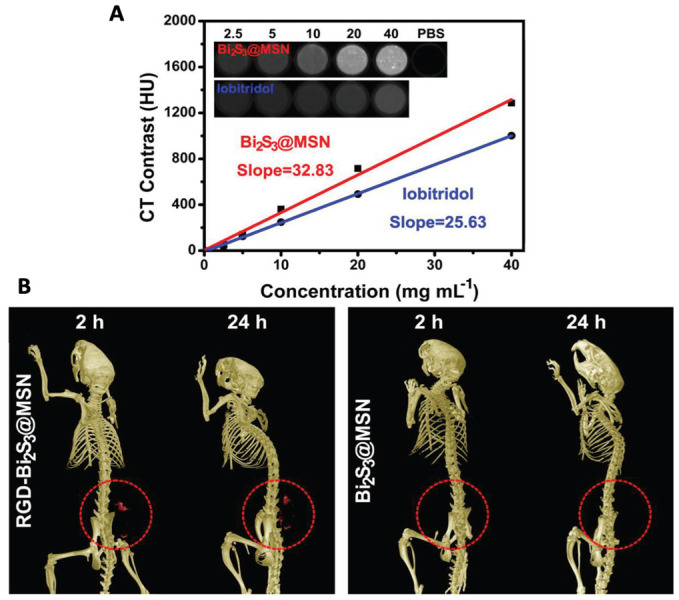
(**A**) In vitro CT performance of Bi_2_S_3_@MSNs in comparison with commercially available iobitridol. Inset: Suspensions of Bi_2_S_3_@MSNs and iobitridol at different concentrations showing CT contrast. (**B**) Representative CT images of UMR-106 tumor-bearing nude mice showing contrast provided by RGD–Bi_2_S_3_@MSN and Bi_2_S_3_@MSN captured 2 and 24 h post-treatment. The red circle highlights the tumor site. Reprinted with permission from Ref. [35]. Copyright © 2023 Wiley.

Nosrati et al. used bismuth sulfide (Bi_2_S_3_-) NPs for combination therapy including chemotherapy and radiotherapy guided by CT imaging [34]. The Bi_2_S_3_-NPs were coated with BSA to improve their stability followed by curcumin encapsulation and functionalization with folic acid to yield Bi_2_S_3_@BSA-FA-CUR NPs. The NPs showed sustained release of curcumin, radio-sensitization effects and a linear increase in CT contrast with increasing Bi concentration. Similarly, Bi_2_S_3_@MSNs, i.e., bismuth sulfide NPs coated with mesoporous silica, were synthesized to enable drug delivery in addition to NIR-responsive PTT and CT imaging [35]. The presence of mesoporous pores in silica enabled high drug loadings up to 99%, whereas the presence of Bi resulted in a high photothermal conversion efficiency of 37%. Figure 2A shows the in vitro CT performance of Bi_2_S_3_@MSNs showing a linear increase with increasing Bi concentration [35]. As can be seen in the figure, the slope of iobitridol (25.63 HU L g^−1^) is lower than that of Bi_2_S_3_@MSNs (32.83 HU L g^−1^). In vivo, the authors evaluated the CT contrast to assess the active targeting potential of RGD (targeting ligand containing arginine(R)-glycine(G)-aspartate(D) triad) conjugated Bi_2_S_3_@MSNs. RGD–Bi_2_S_3_@MSNs show a good accumulation at the tumor site resulting in an increased CT signal from 2–24 h post-i.v. injection as compared to Bi_2_S_3_@MSNs (Figure 2B).

Wang et al. reported the synthesis of hydrophobic Cu_3_BiS_3_-NPs and their use for targeted photodynamic/photothermal therapy and CT/MR dual modal imaging [36]. Modifications to the NPs included coating with DSPE-PEG/DSPE-PEG-NH_2_ (DSPE: 1, 2-Distearoyl-*sn*-glycero-3-phosphoethanolamine-Poly (ethylene glycol)) for hydrophilicity, conjugation of photosensitizer chlorin e6 (=Ce6) and functionalization with folic acid for targeting. The X-ray co-efficient value of Cu_3_BiS_3_-NPs was calculated as 17.7 HU mmol Bi/L, whereas r_1_ relaxivity was found to be twice that of Gd-DTPA, which is a clinically used T_1_-MRI contrast agent. In vivo, these translated into significant CT and MR contrast which peaked at 4–6 h post-i.v. injection via the tail vein. For MRI, a 281.6% increase in signal intensity was observed 6 h post-injection, whereas a quantitative CT value of 252.3 ± 25 HU was observed. Combined, the NPs were able to successfully accumulate at the tumor site and inhibit tumor growth in vivo [35]. In addition, Wang et al. discussed the use of rhenium disulfide (ReS_2_-) NPs as gastrointestinal (=GI) tract and tumor imaging probes, due to their excellent X-ray and NIR absorption properties [37]. With respect to GI tract imaging, the ReS_2_-NPs showed a higher signal-to-noise ratio with increasing X-ray energy 5 min post-oral administration in Kunming mice when compared to iohexol. Similar results were also observed in 4T1 tumor-bearing mice, when ReS_2_-NPs were injected intratumorally, whereby the HU value increased from 30–50 to 110–150 in the tumor region [38].

### 2.3. Optical Imaging

When light is used to probe molecular and cellular interactions for visualization, it is called optical imaging [39]. Depending on the tissue composition, when light travels through it, photons may experience absorption, reflection or scattering. These interactions can be analyzed in different types of optical imaging techniques to yield unique spectral signatures [40]. For example, inelastic scattering of light is measured by Raman spectroscopy, whereas absorption followed by emission of light can be in fluorescence [19]. Optical imaging offers advantages such as the ability to image at the microscopic level and good spatial resolution but is limited by scattering of light in biological tissues. This is often overcome using imaging probes in the NIR region as there is lower absorption and scattering by soft tissue [40].

NPs exploited for optical imaging mostly include QDs, as their emission is often a function of their size and can be effectively tuned. Changes in the size of nanoparticles also leads to changes in their band gap which in turn influences their imaging properties. Optical bandgap, especially of semiconductor materials is inversely proportional to nanoparticle size distribution. Thus, size of QDs often plays an important role in imaging applications. The ability of M_x_S_y_-NPs to absorb in the second biological window, i.e., NIR-II (1000–1700 nm), thus enabling deep tissue penetration, better signal-to-noise ratio with reduced tissue auto-fluorescence has led to their widespread application in optical imaging [41]. M_x_S_y_-NPs studied for optical imaging include semiconductor metal-based QDs especially from group II–VI elements of the periodic table of the elements such as cadmium sulfide (=CdS) and zinc sulfide (=ZnS), respectively. Group I–VI semiconductor-based silver sulfide, i.e., Ag_2_S-NPs are also being increasingly used in optical imaging due to properties like absorption in the second NIR window, high signal-to-background noise ratio and good resolution [42]. Examples of M_x_S_y_-NPs used for different types of optical imaging techniques are reported below.

**Figure 3:**
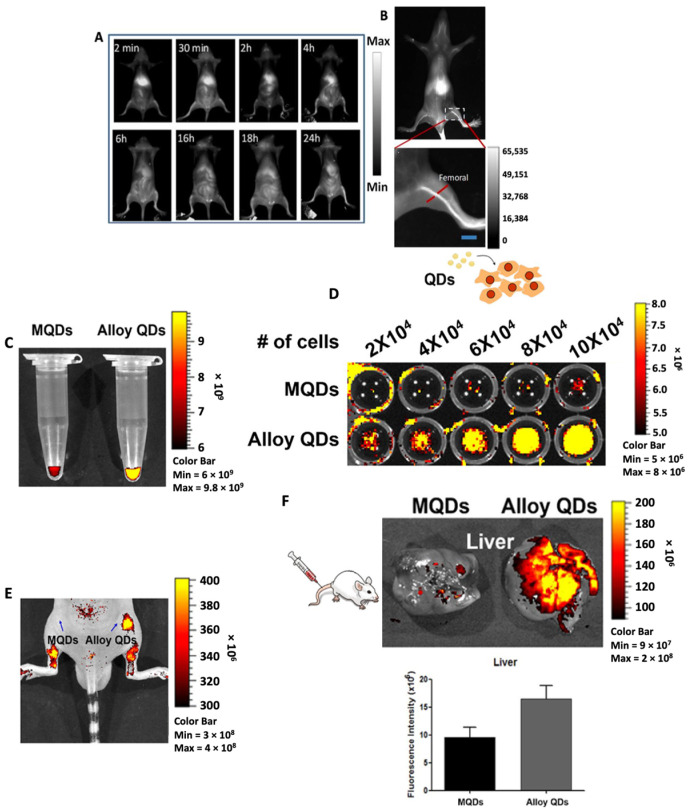
Representative images of NIR-II fluorescence imaging in BALB/c mice. (**A**) Full body distribution and (**B**) zoomed in image showing fluorescence from the femoral artery post-i.v. injection of Ag_2_S QDs. [Reprinted with permission from Ref. [43]. Copyright © 2023, Royal Society of Chemistry]. (**C**) Higher fluorescence intensity observed from the Eppendorf tube containing the same concentration of alloy QDs as compared to MQDs. (**D**) Cell number dependent increase in fluorescence intensity observed in HeLa cells treated with alloy QDs as compared to MQDs. (**E**) Higher fluorescence intensity observed in vivo in mice treated with alloy QDs. (**F**) Images of liver captured 1 h post-treatment showed higher fluorescence in mice treated with alloy QDs. Reprinted with permission from Ref. [44]. Adopted from BioMed Central 2022.

Awasthi et al. prepared Ag_2_S QDs for fluorescence imaging due to their favorable properties including high quantum yield, good photostability and biocompatibility [43]. To improve hydrophilicity and dispersion of the Ag_2_S QDs, they were encapsulated in a PEGylated dendrimer to yield PEG-PATU-Ag_2_S QDs [43]. When excited with a laser at 785 nm, the appropriate QDs exhibited fluorescence at 1110 nm and intensity of fluorescence improved when the QDs attained sizes greater than 25 nm. The authors also prepared A549 cancer cells labeled with Ag_2_S QDs and intravenously injected them into BALB/c mice to test in vivo tracking ability of the QDs. As can be seen from Figure 3, 2 min post-administration, fluorescence signals were observed mainly from the liver which gradually decreased over time. About 30 min following administration, fluorescence signals spread throughout the body, thus showing the distribution of tumor cells in vivo. To probe the ability of Ag_2_S QDs as a vascular imaging agent, PEG_1000_ was used for modification of the QDs followed by i.v. injection into BALB/c mice. After a few seconds post-administration, the main vascular system of the mouse was clearly visible using a real-time monitoring system (Figure 3D).

Recently, silver/silver sulfide Janus NPs (=Ag/Ag_2_S JNPs) for hydrogen peroxide (=H_2_O_2_) triggered NIR-II fluorescence imaging were reported by Zhang et al. [45]. In the presence of H_2_O_2_, the fluorescence of Ag/Ag_2_S JNPs will be “turned on”, whereas in its absence a nearly quenching effect was observed. This mechanism is attributed to an inhibited electron transfer between plasmonic Ag to semiconductor Ag_2_S in the JNP when treated with H_2_O_2_ thus giving rise to electron deficient fluorescent Ag_2_S. Because of the influence of H_2_O_2_ on plasmonic Ag, changes in morphology induced in the Ag/Ag_2_S JNPs post-treatment by H_2_O_2_ was assessed. Ag/Ag_2_S JNPs of size ~15 nm showed a decrease in size to ~10 nm which was in accordance with the mechanism wherein addition of H_2_O_2_ led to oxidation and eventual etching of plasmonic Ag in the JNP [46]. The authors also studied the increase in fluorescence intensity of Ag/Ag_2_S JNPs treated with H_2_O_2_ and observed a 6-fold increase 24 h post-treatment. To confirm that fluorescence arises from the Ag_2_S component, Ag and Ag_2_S NPs were incubated separately with MCF-7 cells. An “always on” signal was observed in the cells in contrast to an “always off” signal solely with Ag NPs. To determine the in vivo H_2_O_2_-triggered fluorescing ability of Ag/Ag_2_S JNP, they were injected intravenously in an AILI mice model of injured liver. PBS- and only Ag_2_S NP-treated groups were chosen as control groups for the study. Whereas the Ag_2_S-NP-treated group showed fluorescence that was “always on”, Ag/Ag_2_S JNP treated mice showed a gradual switch from off to on fluorescence signals with progressing liver injury. Harish et al. synthesized CdS QDs coated with the biopolymer chitosan to improve its stability and biocompatibility [47]. To test the effect of the chitosan coating, the viability of coated and bare CdS QDs were tested in human Jurkat and erythrocyte cell lines. A reduced cytotoxicity of chitosan-coated CdS QDs was found, as compared to the same concentration of solely CdS. Moreover, it was reported that coated QDs were readily taken up by cells as observed by fluorescence imaging analysis. Biocompatibility and uptake of chitosan-coated CdS QDs was attributed to reduced leaching of Cd^2+^ ions from the respective QDs leading otherwise to cytotoxic effects. In the presence of chitosan, released Cd^2+^ ions form coordination bonds with the amino groups of chitosan thus preventing contact with the cells. In another study, Xu et al. generated two cadmium telluride/cadmium sulfide (=CdTe/CdS) core-shell QDs emitting at 545 nm and 600 nm, respectively, to visualize distribution of two chemotherapeutic drugs in a tumor [48]. Coating of CdS over the core resulted in improved quantum efficiency, fluorescence lifetime, stability and biocompatibility of the QDs. The 5-Fluorouracil (=5-FU) and tamoxifen (=TAM) were encapsulated into CdTe/CdS QDs emitting at 545 nm and 600 nm, respectively. To test the effect of the drugs on the tumor resistant cell line MDA-MB-231, the authors conducted a set of experiments. In the first set, the cells were incubated only with QDs-5-FU and in the second set, the cells were incubated with QDs-TAM followed by QDs-5-FU. In the first experiment, green fluorescence of QDs-5-FU was observed only on the cell membrane, whereas in the second experiment green fluorescence was observed within the cell with orange-red fluorescence observed on the cell membrane.

An approach to improve the quantum yield for fluorescence imaging results from the accessibility of QDs in an alloyed core/shell structure containing ZnS in ref. [49]. In this study, Shim et al. modified CIS, i.e., CuInS_2_ QDs, to form a ZnS-CIS alloyed core surrounded by a ZnS shell affording ZCIS/ZnS. The authors attributed this improvement to the suppression of defect states and electronic structure evolution which, in turn, increased radiative channels. In a similar study, alloy type core/shell CdSeZnS/ZnS QDs were synthesized by Kim and colleagues for bio-imaging applications [44]. The authors compared the quantum yield of the CdSeZnS/ZnS QDs (=alloy QDs) against conventional multilayer CdSe/CdS/ZnS QDs (=MQDs). For alloy QDs, a 1.5-fold higher quantum yield than that of MQDs was reported which significantly improved both in vitro and in vivo imaging (Figure 3C–F).

### 2.4. Photoacoustic Imaging

Photoacoustic imaging (=PAI) is a type of modified ultrasound imaging modality in which imaging signals are generated through acoustic (ultrasonic) waves caused by the photothermal effects of a PTT agent and can increase the spatial resolution and imaging depth in vivo [50]. The broad absorption by M_x_S_y_-NPs in NIR-I and NIR-II resulting from localized surface plasmon resonance has led to their applications as PTT agents and thus also as PAI contrast [51].

**Figure 4:**
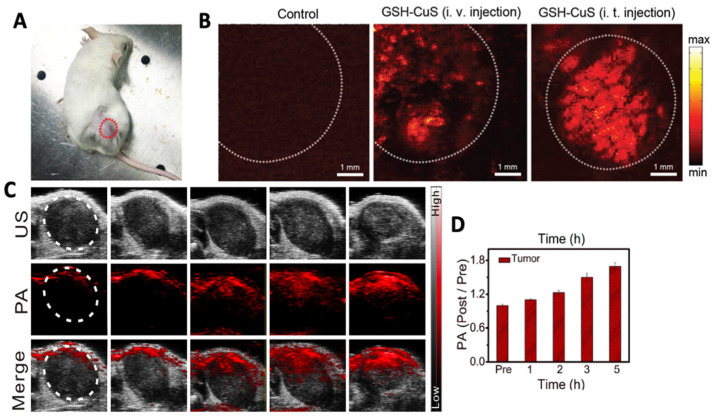
(**A**) 4T1 tumor bearing mice used for in vivo PAI. Laser scan section has been marked with a dotted circle. (**B**) Representative PA images taken before and after GSH-CuS NDs i.v. or i.t. injection Reprinted with permission from Ref. [52]. Copyright © 2023, Royal Society of Chemistry. (**C**) Representative images of PAI and (**D**) its quantitative estimation pre- and post- administration of FeS QDs at different time points. Tumor area has been demarcated with a white circle in (**B**,**C**). Reprinted with permission from Ref. [22]. Copyright © 2023, Elsevier.

Liang et al. prepared glutathione (=GSH)-capped CuS NDs for PTT and PAI via a “one-pot” synthetic methodology [52]. Modification with GSH ensured good water dispersibility and size restriction of the NDs (<10 nm). Under irradiation by a 980 nm laser light, the NDs showed PA contrast three times greater than that of water with a minimal concentration of 1 mM Cu. In vitro studies were followed by in vivo testing in 4T1 tumor-bearing mice. Saline or GSH-CuS NDs were injected intratumorally as control or test, respectively, followed by irradiation at 900 nm. In a control experiment, a very weak PA signal indicating low intrinsic absorption by the tumor at 900 nm, was observed (Figure 4A). On the other hand, a good PA signal was observed in mice treated with GSH-CuS NDs with higher contrast observed in the intratumorally injected mice as evidenced by the enhanced permeation and retention (=EPR) effect and GSH coating on the surface of the NDs. Biomimetic CuS nanoprobes coated with a melanoma cell membrane (HCuSNP@B16F10) for PAI were made accessible by Wu et al. [53]. They loaded HCuSNP@B16F10 with indocyanine green (=ICG) and doxorubicin (=DOX) for PTT and chemotherapy studies. Cell membrane coating was confirmed by Western blotting, and cell viability remained 70% after incubation with 150 µg mL^−1^ for 24 h. In vivo HCuSNP@B16F10 showed a significant PA signal up to 4 h after i.v. injection. In another study, Ouyang and colleagues fabricated CuS nanoparticles trapped in a dendrimer functionalized with PEGylated-RGD (=RGD-CuS DENPs) peptide for PAI-guided PTT/gene therapy [54]. UV–Visible spectroscopy analysis showed good absorption by RGD-CuS DENPs in the 1000–1100 nm range with the CuS core having a diameter of 3.2 nm. The nanoparticles showed PAI contrast dependent on Cu concentration wherein PA signal peaked at 12 h post-intravenous injection in vivo. Figure 4C,D represent PAI obtained using FeS QDs fabricated by Yang et al. which shows a gradual increase in PAI contrast in vivo post-treatment with the QDs.

In addition to X-ray absorption studies, strong NIR absorption has resulted in the application of Bi_2_S_3_ NPs for PAI as well. In this respect, Zhang et al. synthesized hollow Bi_2_S_3_ nanospheres with urchin-like rods (=U-BSHM) for spatio-temporal controlled drug release and PTT-PAI [55]. This was achieved by encapsulating the phase change material (=PCM) 1-tetradecanol and doxorubicin within the microspheres. Heat generated by U-BSHM-NPs under irradiation using an 808 nm laser melted the PCM, which in turn led to the release of DOX thus achieving controlled release. The authors reported a 65.37% release of DOX when U-BSHM-NPs attained a temperature of 43 °C or higher under laser irradiation. With respect to imaging, the NPs showed a concentration-dependent increase in the PA signal intensity by 808 nm laser irradiation. A significant PA signal was also observed when the NPs were irradiated with 700 and 900 nm lasers, respectively (Figure 4A). Zhao et al. synthesized ultra-small Bi_2_S_3_-NPs using self-assembled single-stranded DNA as a template and employed them imaging probe in myocardial infarction [56]. As a result, thereof, a good PA signal was found when tested in vivo. Similarly, Cheng et al. synthesized Bi_2_S_3_ nanorods (=NR) for PTT, radiotherapy, and dual modal PA/CT imaging [11]. In vivo, a significant PA signal post-i.v. injection of the NRs, which peaked 24 h post-treatment, was observed. With respect to CT imaging, the NRs showed an enhanced contrast as compared to the commercially available radiocontrast agent iopromide. The authors concluded that radiotherapy and PTT acted in synergism which inhibited tumor growth as well as metastasis. AgBiS_2_-NDs coated with polyethyleneimine (=PEI) were developed by Lei and colleagues for theranostic applications such as PTT and dual modal PA/CT imaging [57]. PEI-AgBiS_2_-NDs showed photothermal conversion efficiency of 35.2% which translated to a good PAI signal in vitro. With respect to CT imaging, the authors reported a slope higher than that of iobitridol which is a commercially available radiocontrast agent. The respective in vitro imaging results were correlated with in vivo observations and maximum signal intensity for CT/PA imaging was observed at 24 h post treatment.

MoS_2_ which has an extinction co-efficient higher in comparison to gold nanorods (=AuNR) and a 7.8-fold higher NIR absorbance than that of graphene oxide is increasingly being used as an NIR absorbing probe with implications in biomedicine [58]. In order to improve the serum stability of MoS_2_, Shin and colleagues synthesized hyaluronate (=HA) and MoS_2_ conjugates (=HA-MoS_2_) for PAI-guided PTT [59]. The size of MoS_2_ nanoparticles increased from 61.9 nm to 85.9 nm after conjugation with HA. DLS studies revealed no significant changes in the mean hydrodynamic size of HA-MoS_2_ after 7 days in comparison to MoS_2_ alone, indicative of no aggregate formation and, thus, good stability. Liu et al. synthesized MoS_2_ nanosheets conjugated with the dye ICG [60]. The conjugation led to a red shift in the absorption peak of MoS_2_ from 675 nm to 800 nm for MoS_2_-ICG. As a result, a 1.35- and 1.55-fold increase in signal intensity and signal-to-noise ratio were observed at 800 nm pulsed irradiation as compared to that of 675 nm, respectively. The improved PA signal intensity and penetration depth is explained to reduced tissue scattering and absorption at 800 nm. In another study, Au et al. developed nerve growth factor (NGF) targeted AuNR coated with MoS_2_ nanosheets (=anti-NGF-MoS_2_-AuNR) for PAI of osteoarthritis [61]. MoS_2_ coated AuNR resulted in a 4-fold increase in PAI signal intensity and higher biocompaibility as compared to AuNR alone. Additionally, the authors also reported stable PA intensity and morphology of MoS_2_ coated AuNR following irradiation for 30 min. In vivo when anti-NGF-MoS_2_-AuNR were injected intravenously into Balb/c mice, PA signal peaked at 6 h post-treatment in the synovium of osteoarthritic knee. MoS_2_ nanosheets modified with CuS nanoparticles were developed by Zhang and co-workers for PAI-guided chemo-PTT [62]. Colloidal stability and biocompatibility of the nanocomposites were improved by attachment of PEG-thiol (=PEG-SH). CuS-MoS_2_-SH-PEG showed photothermal conversion efficiency higher than that of MoS_2_ alone.

## 3. Applications of Metal Sulfide Nanoparticles in Photo- and Immuno-Therapy

### 3.1. Photothermal Therapy

Photothermal therapy (=PTT) is a non-invasive therapeutic strategy that uses photo-absorbents in the NIR region to induce hyperthermia (40–45 °C) in the tumor site. The NIR laser induces collateral thermal damage to the cancerous cells leading to cell death by apoptosis or by altering gene expression in cancerous cells [63].

**Figure 5:**
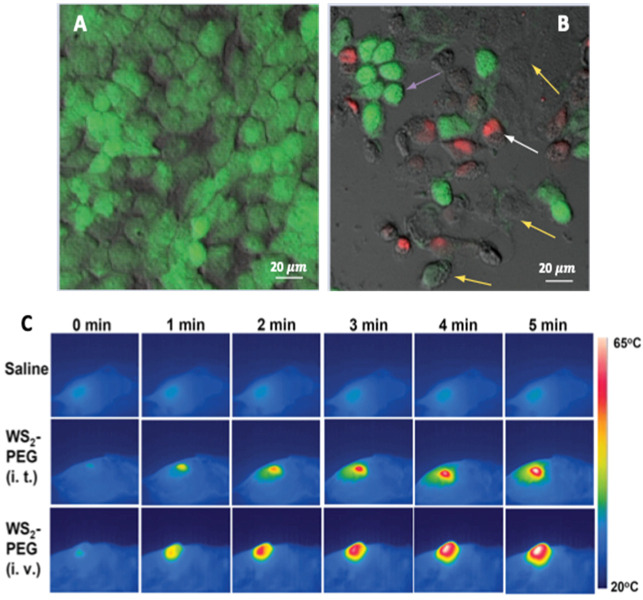
Microphotographs of HeLa cells with CuS-NPs. (**A**) without laser, cells were viable and had polygonal morphology. (**B**) with NIR laser irradiation at 24 W cm^−2^ for 5 min (purple arrows show shrinking of cells; yellow arrows show loss of cell viability by calcein-negative staining; white arrows show loss of cell membrane integrity by EthD-1 positive staining. (**C**) In vivo PTT in 4T1 tumor bearing mice with saline (top row), WS_2_-PEG (middle row: i.t. low dose = 2 mg kg^−1^), WS_2_-PEG (bottom row: i.t. high dose = 20 mg kg^−1^). The laser power density was 0.8 W cm^−2^. Reprinted with permission from Refs. [64,68]. Copyright © 2023, Future Medicine and 2014, Wiley.

CuS-NPs are an emerging class of photothermal agents that are biocompatible, have high extinction in the NIR range, are stable under laser irradiation and, are therefore considered to be better suited than the so far used gold (Au-) NPs [64,65]. The NIR absorption in CuS-NPs is due to d–d energy band transitions of Cu^2+^ ions and therefore their absorption wavelength remains unaffected by the surrounding biological environment. In one report, 980 nm NIR-light-driven CuS nanoplates were found to inhibit the growth of prostate cancer cells both in vivo and in vitro [66]. Respective CuS nanoplates were injected into the prostate tumor site under ultrasound guidance and PTT was performed. Lu et al. reported a platform for dual cancer therapy (photothermal and chemotherapy) based on PEGylated CuS@mSiO_2_ nanocomposites [67]. The mesoporous silica allowed high payload capacity; however, this showed poor colloidal stability. Hence, polyethylene glycol grafting was carried out to improve the colloidal stability and enhance the EPR effect to deliver drugs to the target cells. Cheng et al. developed WS_2_ nanosheets as PTT agent for bio-imaging and photothermal ablation of tumors [68]. The nanosheets were functionalized with PEG to enhance physiological stability and biocompatibility. The 4T1 cells were incubated with 0.1 mg ml^−1^ WS_2_-PEG nanosheets for 6 h and irradiated by an 808 nm laser of varying power densities. The nanosheets effectively induced thermal ablation at a low dose (i.t., 2 mg kg^−1^) and a higher dose (i.v. injection, 20 mg kg^−1^) without causing any mortality (Figure 5). On similar lines, PVP-functionalized MoSe_2_ nanosheets in a PNIPAM hydrogel with both a dual photo- and thermo-responsive behavior was effective towards HeLa cells [69]. Photo-thermal ablation of mammalian cells was also demonstrated by Chou et al. by using chemically exfoliated MoS_2_-NPs at a very low concentration (<38 ppm) to effectively destruct the cancerous cells [70].

Qian et al. introduced PEGylated titanium disulfide (=TiS_2_) as PTT agent for in vivo PAI-guided thermal ablation of cancer [71]. PEG was incorporated into the system to make the nanoparticles stable in polar solvents. The PTT agent exhibited strong NIR absorbance being able to destroy tumor cells. A multifunction theranostics platform, based on WS_2_ QDs (3 nm), was synthesized to achieve simultaneous CT/PAI and synergistic PTT treatment of tumors, wherein the location of the tumor could be precisely observed and treated [72]. MRI-guided PTT was reported using iron sulfide nanoplates. Yang and coworkers prepared PEG-functionalized FeS nanoplates (=FeS-PEG) that exhibited high NIR absorption and superparamagnetism [73]. Highly effective in vivo PTT ablation in mice tumor was achieved using 20 mg kg^−1^ of FeS-PEG followed by 808 nm laser irradiation. MRI studies revealed accumulation of FeS-PEG NPs in the tumor cell and no toxicity was observed even at a higher dose.

Though metal sulfide NPs can be effectively used for photothermal ablation, however, poor photothermal conversion efficiency restricts their use for all practical applications. To overcome this limitation, a combination of metal/metal sulfide NPs was designed [74]. Yang et al. reported surface plasmon-enhanced PTT using an Ag/CuS nanocomposite for effectively killing PC3 prostate cancer cells [75]. The nanocomposite was activated by a 980 nm laser at 0.6 W cm^−2^ for 5 min and, hence, an enhancement of CuS PTT efficacy was observed. This is attributed to the presence of surface plasmon resonance (=SPR) of the Ag-NPs that led to significant enhancement in the electric field near the surface, thereby increasing the rate of the transition process at the interfaces. Ding and coworkers studied the influence of dual plasmonic Au-Cu_9_S_5_-NPs on the photothermal transduction efficiency [76]. The nanocomposite exhibited localized SPR in both the visible and NIR region and the molar extinction coefficient of the composite was found to be 50% higher at 1064 nm than the individual counterparts. The composites were used for PTT on tumor-bearing mice at 100 ppm under 0.6 W cm^−2^ 1064 nm laser irradiation. Similar observations were reported by tuning localized SPR by applying Cu_5_FeS_4_-NPs to enhance the photothermal conversion efficiency up to 50.5% using an 808 nm laser [77].

### 3.2. Photodynamic Therapy

Photodynamic therapy (=PDT) is a clinically approved minimally invasive therapeutic modality in which a photosensitizer (=PS) is activated by a light of specific wavelength (laser) to generate singlet oxygen species (^1^O_2_) that destroys abnormal cells [78]. When the photosensitizer is excited, it transfers its energy to the molecular oxygen in tumor cells through a triplet state. During the process, cytotoxic singlet oxygen and other secondary molecules such as reactive oxygen species, super-oxides, etc., are formed via oxidation of cellular macromolecules. This event leads to necrosis or apoptosis of tumor cells [79]. Nanoparticles can be used as carriers of PS due to (i) easy functionalization with target molecules that increases biodistribution of PS, (ii) the higher surface area-to-volume ratio of NPs increasing the carrying capacity of PS, (iii) protect degradation of light-sensitive PS and enhance their circulation in bloodstream, and (iv) capability to incorporate other therapeutic or diagnostic modalities to PDT in the same system. M_x_S_y_-NPs have an edge over other NPs such as gold NPs for use in PDT, due to their strong absorption properties in the NIR region ranging from 700–1100 nm, high extinction coefficients and high fluorescence properties. Hence, the following sections will focus on M_x_S_y_-NPs that are widely applied in PDT.

Jia et al. used MoS_2_ nanoplates for fluorescence imaging of ATP and PDT through ATP-mediated controllable to release ^1^O_2_ under 660 nm laser irradiation [80]. Therefore, Ce6-aptamer was loaded on the MoS_2_ nanoplates that especially responded to the ATPs in lysosomes and ^1^O_2_ induced cell death through the lysosomal pathway. The studies exhibit the release of a single-stranded aptamer from the MoS_2_ nanoplates and subsequent imaging of intracellular ATP and generation of singlet oxygen.

Plasmonic Cu_2−x_S-NPs confirmed excellent surface plasmon absorption in the NIR region which mainly originates from the free holes of the unoccupied highest energy state of the valence bond [81]. This depends on the ratio of Cu:S and the crystal phase of the nanoparticles itself. Examples of plasmonic Cu_2−x_S-NPs are Cu_31_S_16_ (monocyclic phase), Cu_9_S_5_ (cubic phase), Cu_7_S_4_ (orthorhombic phase), Cu_58_S_32_ (triclinic phase), and CuS (hexagonal phase or covellite). With decrease in the Cu:S ratio (Cu_2−x_S with x > 0), the concentration of free carriers increases inducing LSPR absorbance in the NIR area. Cu_2−x_S-NPs enhanced the ROS generation in B16 cells under NIR radiation (808 nm, 0.6 W cm^−2^ for 5 min) [82]. Generation of hydroxyl radicals were detected by 5,5-dimethyl-1-pyrroline-N-oxide (=DMPO) spin-trapping adducts in electron spin resonance (=ESR) spectroscopy. The ROS generation was dependent on the concentration of the NPs and the laser power. From the ESR signal it can be concluded that the irradiation led to around 83% enhancement in •OH generation. In vivo, Cu(II) is reduced to Cu(I) by biomolecules such as ascorbic acid or glutathione, which reacts with hydrogen peroxide to form •OH species. Similar results were also obtained by other researchers [81,83,84,85].

Cheng and coworkers reported on the use of Bi_2_S_3_ nanorods for NIR-activated PDT [86]. The nanorods could be excited by a NIR laser to generate free holes in the valence band and electrons in the conduction band, which formed hydroxyl and superoxide radicals upon reaction with water and oxygen. Further, when the nanorods were associated with zinc protoporphyrin IX, a pronounced inhibitory effect of the tumor was observed under NIR irradiation. Lin et al. synthesized Co_9_S_8_ NDs and modified their surface with albumin to make them biocompatible [67]. Upon NIR irradiation, the NDs showed a marked time-dependent production of ^1^O_2_ production with high photothermal conversion efficiency of 64%.

### 3.3. PTT-PDT Combinatorial Therapy

Photothermal and photodynamic therapies have an edge over conventional therapies including chemotherapy, surgery, and radiation due to high specificity, minimal invasion, and precise spatio-temporal selectivity [87]. Furthermore, in PTT and PDT, no extra targeting is required, however, tissue penetration of light is a concern. Heat conversion efficiency and formation of hypoxic environments in PTT and PDT are other concerns. PTT agents convert light energy into heat and eradicate tumors by hyperthermia, while PDT agents produce toxic reactive oxygen species to kill cancer cells. However, PTT generally requires high-power density lasers to produce enough heat and PDT requires the effective uptake of photosensitizers by cancer cells to induce tumor hypoxia. In other words, in PTT, self-protection of cancer cells induces heat shock response which weakens the PTT efficacy and on the other hand, in PDT, tissue hypoxia limits the PDT efficacy. Therefore, synergistic strategies by combining PTT and PDT in a single platform are now becoming important to overcome the concerns and gain improvised results of the therapies. Simultaneous hyperthermia and ROS are envisaged to cause cancer cell death and elimination of malignant tumors by PTT-PDT combinatorial therapy. In such cases, a single nanoplatform that can behave as both PTT and PDT agents are highly desirable. Following section deals with metal sulfide nanomaterials that are visualized to be PTT as well as PDT agents.

**Figure 6:**
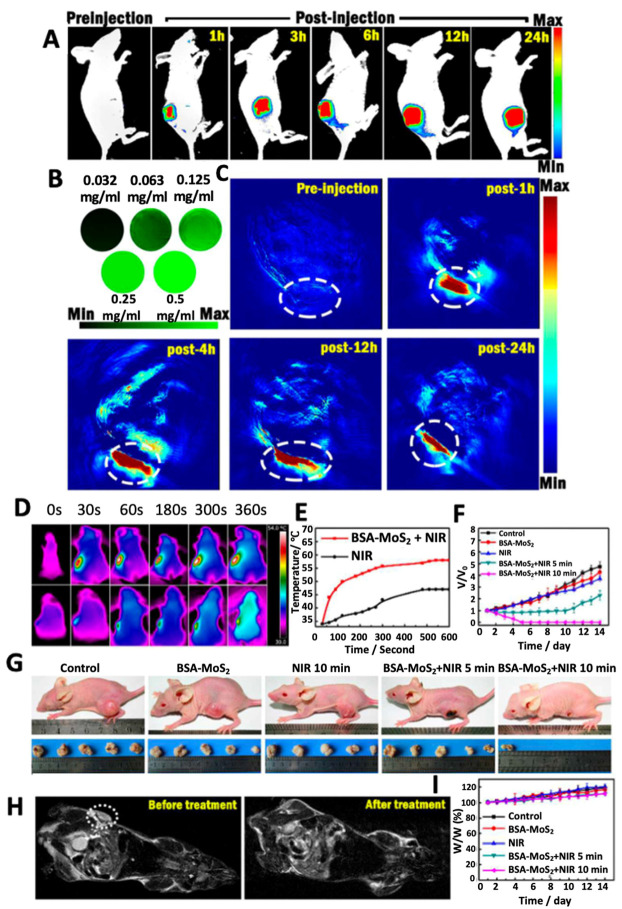
(**A**) Representative fluorescence images of BSA-MoS_2_ treated tumor-bearing mice at different time points. (**B**) In vitro PAT imaging for different concentrations of BSA-MoS_2_. (**C**) Representative PAT images of BSA-MoS_2_ treated tumor-bearing mice at different time points with tumor area marked with a white dotted line. (**D**) Representative infrared images showing thermal profile of tumor-bearing mice treated with BSA-MoS_2_ or PBS (control group) and their corresponding (**E**) temperature profile and (**F**) tumor volume. (**G**) Representative pictures of mice showing reduction in tumor size with respective treatments. (**H**) MR images of mice treated with BSA-MoS_2_ before and post- treatment on the 14th day and corresponding (**I**) changes in body weight. Reprinted with permission from Ref. [88]. Copyright © 2023, Royal Society of Chemistry.

Song et al. designed bioconjugated MoS_2_ nanosheets for combinatorial PTT-PDT in which bioconjugation was done with BSA to render biocompatibility to the nanosheets (Figure 6) [88]. The bioconjugated nanosheets produced both localized hyperthermia and ^1^O_2_. A possible mechanism of the combinatorial effects can be explained by the following route: Firstly, when BSA-MoS_2_ nanosheets are irradiated with an 808 nm laser at 0.8 W cm^−2^, a rise in temperature (up to 48 °C in 4–5 min) takes place which then activates the dissolved oxygen to generate ROS (in the order O_2_ → ^1^O_2_ → O_2_^•−^ → ^•^HO_2_ → H_2_O_2_ → ^•^OH). Thus, BSA-MoS_2_ nanosheets trigger ROS generation and enhance the phototherapy. In another study, following a similar mechanism, MoS_2_ nanosheets in hydrogel were used as PTT and PDT agent along with chemotherapy [89]. Remarkable reduction in primary 4T1 breast tumors and distal lung metastatic nodules in vivo was observed. A mild photothermal heating was able to increase cell membrane permeability and cellular uptake of various agents such as photodynamic agents or chemotherapeutic drugs [90]. Similar results were obtained by Xu and his group wherein an IR-808 dye sensitized UCNP with Ce60-grafted MoS_2_ nanosheets synergistically amplified the up-conversion efficiency and triggered the photosensitizer to produce large amounts of ROS [91].

The combination of PDT and PTT was also demonstrated by Bharathiraja and coworkers where MBA-MD-231 cells were incubated with CuS-Ce6 NPs and exposed to an 808 nm laser light for 10 min at 2 W cm^−2^ [92]. MTT assay revealed synergistic cytotoxicity by the combination therapy rather than individual therapies. Similar observations were made by Wang’s group [63]. Heat generation, due to photothermal efficacy of Cu_2−x_S-NPs, was monitored in B16 cells by heat shock protein 70 (Hsp70) expression. The cells exposed to 100 s laser radiation (808 nm and 0.6 W cm^−2^) showed significantly enhanced Hsp70 which is caused not only due to thermal stress but also due to elevated ROS levels [82]. Under NIR light and in tumor acidic regions, leaking of Cu(I) ions from the NP occurs, which react with the surrounding O_2_ and H_2_O_2_ to form Cu(II) along with hydroxide and hydroxyl radicals that contribute to enhanced ROS [93]. Biocompatible PEGylated iron sulfide NPs (=FeS_2_@C-PEG) were found to oxidize water to form O_2_ under NIR exposure which improved the therapeutic efficacy of the NPs [94]. Formation of Fe(II) degraded the intracellular H_2_O_2_ to produce more ROS species that contributed to the combinatorial PTT-PDT. Zinc protoporphyrin IX (=ZP)-linked Bi_2_S_3_ nanorods provide active sites for binding heme oxygenase-1 (HO-1) that are overexpressed in solid tumors and suppressing the cellular antioxidant defense capability. The nanorods, upon NIR radiation, generated heat that facilitated an efficient electron–hole separation in ZP and Bi_2_S_3_ and produced ROS species. Once cells are attacked by ROS, the redox homeostasis is disturbed and HO-1 catalyzed the heme molecule to generate a series of antioxidants (biliverdin, carbon monoxide, and ferrous iron), which are the most potent endogenous scavengers of ROS. Here, ZP, as a potent HO-1 inhibitor, suppressed the HO-1 activity and strengthened the PDT effect. Under 808 nm laser irradiation (0.75 W cm^−2^, 10 min), the nanorods exhibited photothermal conversion efficiency of 33.64%. The nanorods could accumulate in the 4T1 tumor and inhibit the HO-1 activity and enhance NIR-irradiated oxidative injury [86]. Cobalt chalcogenides also possess intrinsic peroxidase-like activity, high photothermal conversion efficiency and broad NIR absorption properties; however, it is challenging to synthesize biocompatible cobalt sulfide due to co-existence of both strongly reducible cobalt ions and oxidizable sulfide ions. Further, cobalt ions have strong affinity for oxygen and, therefore, it is difficult to exclude impurities such as cobalt oxide or cobalt hydroxide in the resultant NPs [95,96].

### 3.4. Combined Photo-Immunotherapy

Immunotherapy is a biological cancer treatment that makes use of substances from living organisms to treat cancer and help the immune system to fight cancer. Specifically, immunotherapy or immune activation involves production of cancer-fighting immune cells to identify and destroy cancerous cells. Immunotherapy includes checkpoint inhibitors, T-cell transfer, monoclonal antibodies, cancer vaccines and immune system modulators. In contrast to conventional therapies such as chemotherapy, radiotherapy, or surgery, that aim to destroy cancer cells along with healthy cells, immunotherapy aims to prevent the healthy cells and restore antitumor activity of the immune system. Research on delivery of immunotherapeutic agents by NPs showed minimization of adverse effects and maximization of the therapeutic index of immunotherapy [97]. Nanomaterial-based delivery of immunotherapeutics and biologicals (e.g., nucleic acids, antibodies, etc.) improves pharmacological properties of drugs such as solubility, and stability in physiological media. Assorted molecular-binding sites in nanomaterials help in shielding active drugs and biologics from degradation and macrophage clearance in blood after systemic administration. In other words, nanomaterials enhance bioavailability and control unwanted targeting which is significant in tumor management [98]. Further, the pharmacokinetic profile of the drug and their interaction with cells can also be modulated and controlled by the nanosystem [99]. Release of the drug or biologics can also be controlled and regulated by nanomaterials to enhance efficacy and reduce systemic toxicity. However, it is important to consider the structure and composition of NPs for active targeting of drugs or biologics and their release. Above all, nanotechnology offers possibilities of combining immunotherapy with chemo-, radio- or even photothermal and photodynamic therapies.

Several nanosystems ranging from carbon-, metal/metal oxide-, polymer- and lipid-based NPs are reported for specific delivery of immunotherapeutics to precisely target and control tumors [100,101]. However, very little literature is available on the use of metal sulfide NPs for immunotherapy. The following section will focus on metal sulfide-based NPs that are reported for immunotherapy along with other phototherapies.

Guo and his group designed a light-induced transformative NP platform based on chitosan-coated hollow CuS-NPs that can assemble immunoadjuvants oligodeoxynucleotides containing the cytosineguanine (CpG) motifs [102]. The platform combined photothermal ablation and immunotherapy in which, upon laser excitation at 900 nm, the nanostructures broke and reassembled into polymer complexes which enhanced CpG tumor retention and uptake by plasmacytoid dendritic cells. It generates heat to ablate the tumor cells and releases the tumor antigens into the tumor sites, while the immunoadjuvants enhance antitumor immunity by promoting antigen uptake. The PTT synergistically acted with immunotherapy to enhance immune responses and made the tumor residues and metastases susceptible to immune-mediated killing. Similar observations were made by Chen et al. using core-shell CuS@PLGA-NPs in which the model antigen ovalbumin (OVA) was loaded [103]. On one hand, poly D, L-lactic-co-glycolic acid (=PLGA) made the system biocompatible and exhibit controlled biodegradation kinetics, and on the other hand, the CuS-NPs display favorable PTT by killing 4T1 tumor cells in vitro. Release of OVA and its further internalization into antigen-presenting cells (=APCs) induced the immune response. The heat conversion by CuS-NPs under NIR radiation not only triggered rapid release of OVA but also enhanced the cell membrane permeability that led to higher uptake of the antigen by the cells. Yan et al. reported synergistic PTT and immunotherapy driven by Cas9 ribonucleoprotein-loaded CuS-NPs to enhance the therapeutic effect on melanoma [104]. The NIR light triggered thermoresponsive CuS-NPs provide a platform to modify Cas9 ribonucleoprotein targeting PTPN2 for immunotherapy. Depletion of PTPN2 was observed after treatment with the targeted NPs which caused accumulation of infiltrating CD8 T lymphocytes in tumor mice. Also, the expression levels of interferons and cytokines (IFN-γ and TNF-α) was upregulated which sensitized the tumors to immunotherapy. Thus, tumor ablation along with immunogenic cell death induced by PTT amplified the anti-tumor efficacy. Similar integration of PTT and immunotherapy in a Cu_9_S_5_@mSiO_2_ nanoagent was reported in a study by Zhou et al., in which the immune response of CpG effectively inhibited tumor metastasis [105]. Intracellular uptake of CpG promoted infiltration of cytotoxic T lymphocytes (=CTLs) in tumor tissue, which stimulated the production of IL-12, TNF-α and IFN-γ. Xu and coworkers verified adoptive macrophage therapy through CuS-NP regulation for antitumor effect in mice bearing B16F10 melanoma [106]. Within this study, bone-marrow-derived macrophages (=BMDMs) were incubated with PEGylated CuS-NP to promote cellular production of ROS through dynamin-related protein 1 (Drp1)-mediated mitochondrial fission. The high intracellular ROS level directs BMDMs polarization toward M1 phenotype by classical IKK-dependent NF-κB activation. Moreover, the CuS-NP-stimulated BMDMs downregulated PD-1 ligand expression and contributed to the promoted ability of phagocytosis and digestion. I.t. transfer of CuS-NP-redirected macrophages, triggered the local and systemic tumor-suppressive alterations, further enhancing the antitumor activity. On similar lines, MoS_2_ nanosheets were functionalized with CpG and PEG to form nanoconjugates that upon NIR irradiation significantly enhanced intracellular accumulation of CpG [107]. The accumulation of CpG stimulated the production of proinflammatory cytokines and elevated immune response. The MoS_2_ nanoconjugates also reduced proliferation of 4T1 cells when co-cultured with RAW264.7 (macrophage cells) upon NIR irradiation for 10 min at 2 W cm^−2^. The increased uptake efficiency of CpG is attributed to the membrane permeability induced by laser irradiation.

MoS_2_-NPs are able to induce low levels of the pro-inflammatory cytokines IL-1β, IL-6, IL-8, and TNF-α in human bronchial cells (NL-20) and activate antioxidant/detoxification defense mechanisms [108]. The low cytotoxicity of the MoS_2_-NPs reflects the ability of the NPs to induce a favorable balance of cellular responses in vitro which can be extended to in vivo in future.

It can be inferred that the combination of photothermal therapy and immunotherapy can produce synergistic anti-tumor effects as well as reduce systemic toxicity [109]. Major applications of M_x_S_y_-NPs in photothermal therapy are due to their ability to convert NIR radiation into thermal energy which is subsequently used for ablation of cancer cells. However, it is important to achieve higher conversion efficiency so that the dose requirement is reduced. Moreover, integration of photothermal therapy with immunotherapy is essential to address cancer heterogeneity and adaptation.

## 4. Conclusions

Although M_x_S_y_-NPs have been researched as theranostic nanoplatforms over a decade, only a handful of reviews are highlighting recent developments and challenges in this field [9,10,20]. Metal sulfide NPs, specifically, transition metal dichalcogenides, have an array of desirable properties such as electronic band structure, tunable bandgap, luminescence, and Raman scattering, which can be tuned as per the end applications. However, because of the semiconductor behavior, they are intrinsically toxic which limits their use in biomedical applications. To address the concern, additional modifications of the appropriate nanomaterials are required to enhance biocompatibility and make them capable for their use as diagnostic tools or imbibe properties for applications such as drug delivery, sensing, etc. Further, metal sulfide NPs do not form very stable suspensions in polar solvents, for example, water, and therefore, their use in in vivo applications also remains a concern. Hence, proper NP functionalization is, therefore, required to provide colloidal stability to the respective NPs. Thus, selection of functional molecules (e.g., dyes, polymers, organic molecules including acids, small molecules such as hydroxyl, thiols, etc.) are crucial for facilitating interactions between the NPs and biological systems [110]. In many cases, functionalization may involve modification of atoms of the NPs present in the basal plane, kinks, edges or corners, which may change the electronic band structure of the NPs [111]. Voiry et al. reported that change in phase of sulfur- and selenium-based transition metal dichalcogenides from metal to semiconductor takes place when the NPs will be covalently functionalized with, for example, amides and methyl moieties, respectively [112]. Thus, designing synthesis and functionalization strategies of metal sulfide NPs are very important to meet the requirement of structural and chemical stability, dispersibility in physiological medium, uniformity in size distribution, and biocompatibility. In addition to functionalization, core–shell structures may also be developed to decrease leaching of toxic metals in cellular environments. This is especially true in heavy metal quantum dots such as lead sulfide (PbS), CdS, mercuric sulfide (HgS) offering excellent optical imaging properties but are limited due to their cytotoxicity. In such cases, formation of a shell over the core can impede direct contact of the heavy metals with cells and improve biocompatibility of the appropriate metal sulfide.

Though multimodal platforms (therapeutic and imaging) have proved beneficial for treatment of several diseases, overtreatment is emerging as a new concern. Minimizing the use of probe material and therapeutic dose, while maintaining the effectiveness of the platform, is crucial for patient’s compliance. Integration of various functions in a nanosystem without changing individual properties can significantly synergize theranostic effects. It is also important to design a multimodal system of varying chemistries that would not only retain their individual functions, but also not interfere with the functions of other materials, which eventually can enhance the effectiveness of every component. Li et al. developed such a platform based on hydrophilic MnS@Bi_2_S_3_-PEG NPs which was successfully used as contrast agents for MRI, CT and PA-trimodal imaging moiety along with PTT and hyperthermia applying a single injection dose for tumor therapy. Hyperthermia significantly enhanced the efficacy of radiation and provided a unique platform to address the concern of overtreatment [113]. More such platforms would definitely prove beneficial; however, their short- and long-term efficacies and toxicities need to be evaluated.

Nanoparticle-based delivery of immunotherapeutics is significant in not only treating cancer but also developing immune defensive cells that can be used to identify and eliminate tumor cells. Due to limited toxicity and side-effects, immunotherapy can be used in conjunction with other interventions such as chemotherapy, radiation therapy, photothermia and hyperthermia. Several multifunctional nanomaterials have been explored as photoimmunotherapeutic agents to enhance phototherapy as well as carrier of immune adjuvants. Despite the progress, more research is required to understand the dynamic immune response and the molecular mechanism of NPs-immune interaction for promoting clinical translation of nano-immunotherapy. It is also important to consider the potential risk associated with overstimulation of the immune system that may lead to autoimmune toxicities. A balance between efficacy and safety rather than a strong anti-tumor immune response is required. Nevertheless, photoimmunotherapy has shown promising pre-clinical responses on various tumor models and therefore, has a potential for clinical translation.

Though there are proven reports of the versatility of M_x_S_y_-NP-based nanophototherapeutic platforms, clinical translation is a long way to go. More detailed understanding of degradations and metabolism of M_x_S_y_-NPs is required to validate their effectiveness with respect to degradation products of M_x_S_y_-NPs, metal metabolism, biodistribution, pharmacokinetic mechanism, fate, and elimination process. Nevertheless, advancements in research will have an impact on future phototherapeutic abilities of M_x_S_y_-NPs.

## Data Availability

Not applicable.
